# Abnormal neural activation patterns underlying working memory impairment in chronic phencyclidine-treated mice

**DOI:** 10.1371/journal.pone.0189287

**Published:** 2017-12-18

**Authors:** Yosefu Arime, Kazufumi Akiyama

**Affiliations:** Department of Biological Psychiatry and Neuroscience, Dokkyo Medical University School of Medicine, Mibu, Tochigi, Japan; Technion Israel Institute of Technology, ISRAEL

## Abstract

Working memory impairment is a hallmark feature of schizophrenia and is thought be caused by dysfunctions in the prefrontal cortex (PFC) and associated brain regions. However, the neural circuit anomalies underlying this impairment are poorly understood. The aim of this study is to assess working memory performance in the chronic phencyclidine (PCP) mouse model of schizophrenia, and to identify the neural substrates of working memory. To address this issue, we conducted the following experiments for mice after withdrawal from chronic administration (14 days) of either saline or PCP (10 mg/kg): (1) a discrete paired-trial variable-delay task in T-maze to assess working memory, and (2) brain-wide c-Fos mapping to identify activated brain regions relevant to this task performance either 90 min or 0 min after the completion of the task, with each time point examined under working memory effort and basal conditions. Correct responses in the test phase of the task were significantly reduced across delays (5, 15, and 30 s) in chronic PCP-treated mice compared with chronic saline-treated controls, suggesting delay-independent impairments in working memory in the PCP group. In layer 2–3 of the prelimbic cortex, the number of working memory effort-elicited c-Fos+ cells was significantly higher in the chronic PCP group than in the chronic saline group. The main effect of working memory effort relative to basal conditions was to induce significantly increased c-Fos+ cells in the other layers of prelimbic cortex and the anterior cingulate and infralimbic cortex regardless of the different chronic regimens. Conversely, this working memory effort had a negative effect (fewer c-Fos+ cells) in the ventral hippocampus. These results shed light on some putative neural networks relevant to working memory impairments in mice chronically treated with PCP, and emphasize the importance of the layer 2–3 of the prelimbic cortex of the PFC.

## Introduction

One of the critical unmet needs in schizophrenia management is an effective treatment for cognitive impairments [1, 2]. Accumulating evidence indicates that cognitive impairments are present throughout the entire course of the disease, even in the premorbid conditions [3] and at first-onset episodes [4], and endure in later stages of the disease [5, 6]. These impairments are usually accompanied by compromised daily activity and lead to poor long-term outcomes [1, 7]. Hence, novel pharmacotherapies that remedy cognitive impairments in patients with schizophrenia are urgently needed.

Working memory is defined as the ability to transiently maintain and manipulate information to guide goal-directed behavior. Human studies have shown that the prefrontal cortex (PFC) acts as a central hub for such a goal-directed behavior by integrating new sensory information and transiently retrieved long-term memory storage [8, 9]. The importance of the PFC in working memory has attracted considerable attention, and much effort has been devoted to in-depth analysis of PFC dysfunction in the working memory impairments of schizophrenia. However, specific neural circuits underlying working memory impairments in schizophrenia have not been identified [10, 11].

The glutamate hypothesis has provided a substantial contribution to the development of rodent models of schizophrenia. Several lines of evidence have shown that schizophrenia is associated with deficits in N-methyl-D-aspartate (NMDA) glutamate receptor-mediated neurotransmission [12, 13]. Phencyclidine (PCP) and ketamine, representative noncompetitive antagonists of NMDA receptors, induce a wide range of symptoms resembling schizophrenia in healthy individuals, including both positive and negative symptoms as well as cognitive deficits [14–17]. Rodent experiments have shown that repeated PCP administration impairs executive function, working memory, and sociability [18–20], suggesting the utility of this rodent model to investigate neural substrates underlying cognitive impairments associated with schizophrenia [21, 22].

Spatial working memory has been measured in rodents using a range of cognitive tasks, but Pratt and colleagues addressed an important caveat for interpretation of task construct validity [10]. Only tasks that require focused recruitment of PFC would enable a valid comparison of brain regions subserving spatial working memory between rodents and humans. Of note, the delayed non-match to position task has been well validated as a reliable method to measure spatial working memory across species [21]. However, it remains unexplored whether neural circuits subserving delayed non-match to position task are modulated after chronic PCP exposure. Accordingly, further study is warranted to identify brain regions associated with working memory impairments in rodents chronically treated with PCP.

In the present study, we first assessed working memory performance in mice chronically treated with saline or PCP using the discrete paired-trial variable-delay task in T-maze, which was developed by Moghaddam et al. and has been refined as a delayed non-match to position paradigm assessing working memory in both rodents and clinical subjects [23]. Specifically, we used a variant of this T-maze protocol modified for mice. Second, we mapped expression of c-Fos, an immediate early gene (IEG) induced by calcium influx triggered by synaptic inputs [24], across brain regions after completion of the working memory task to analyze group differences in neural activity patterns. This strategy allows for analysis of task-related activation maps with single-cell resolution [25]. Based on the cellular resolution of brain-wide c-Fos mapping, we explored brain regions related to working memory impairment in this model.

## Materials and methods

### Animals

Inbred male C57BL/6J mice (6–7 weeks old) were purchased from Japan Clea Co. (Japan). Mice were housed 2–4 per cage in a temperature-controlled (25 ± 1°C) and light-controlled room (lights on 0600–1800 h) in plastic cages with *ad libitum* access to water and food restriction (described below). All animal experiments were approved by Dokkyo Medical University School of Medicine for Animal Experiments (Permit Number: 0704), performed in accordance with the Guidelines for Care and Use of Laboratory Animals, Dokkyo University School of Medicine, and conformed to all Japanese federal animal welfare rules and guidelines. All efforts were made to minimize animal suffering and to reduce the number of animals used.

### Drug

Phencyclidine hydrochloride (Sigma-Aldrich, UK) was dissolved in saline and administered subcutaneously (s.c.) at 10 ml/kg body weight. The mice were treated with PCP 10 mg/kg or equal-volume saline daily for 14 consecutive days in the home cage.

### Working memory task in T-maze

**Fig 1:**
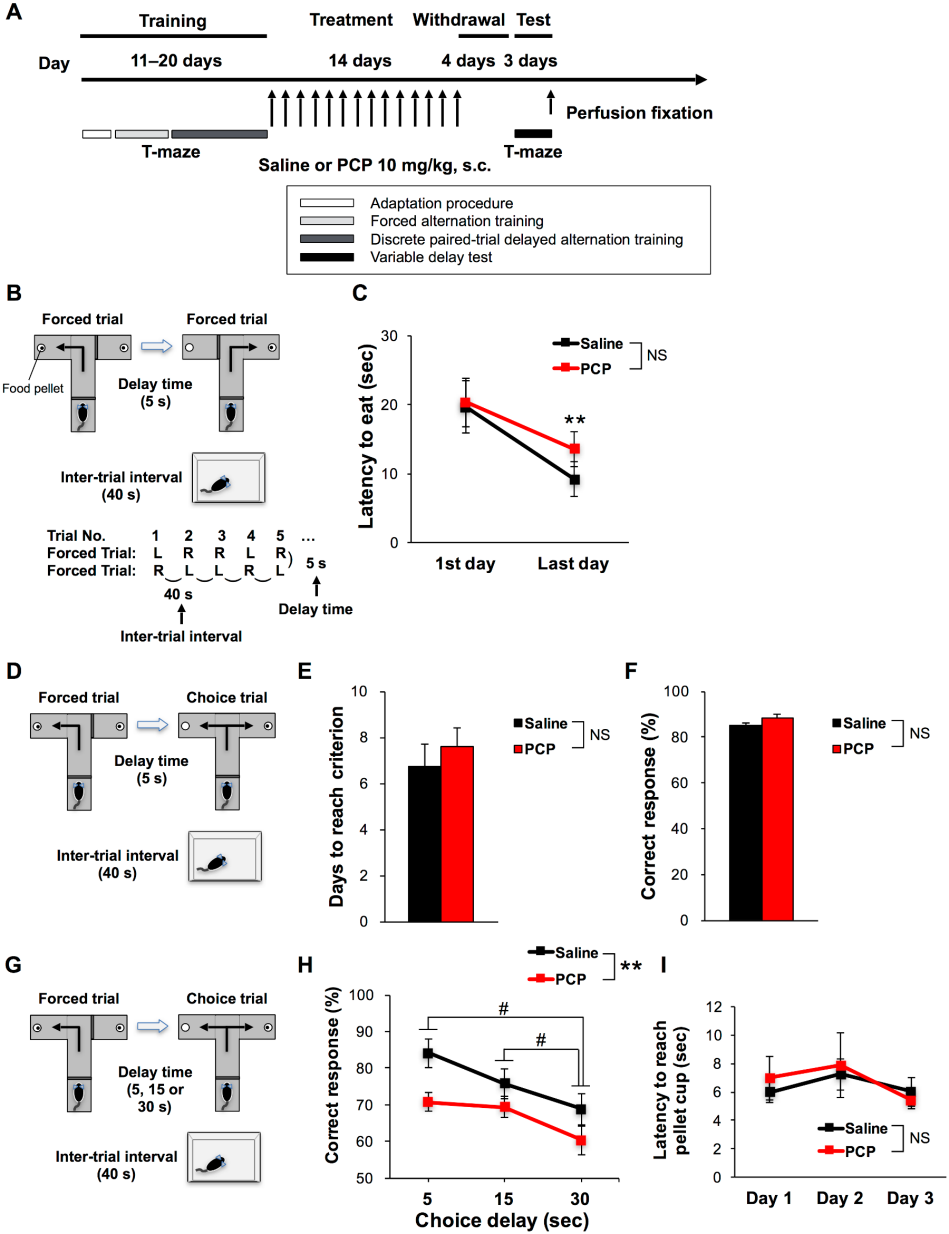
Working memory impairment, which is not delay-dependent in chronic PCP-treated mice. (A) Experimental schedules for the discrete paired-trial variable-delay task in T-maze and the drug treatments. (B) Schematic illustration of the forced alternation training sessions. (C) Significant decreases in latency to eat food pellets were observed over days during the forced alternation training with no group differences. (D) Schematic illustration of the discrete paired-trial delayed alternation training sessions. Before drug treatment, no group differences in days to reach criterion (E) and correct responses (F) were observed. (G) Schematic illustration of the variable delay test in T-maze. (H) Percent of correct responses by chronic saline- (n = 11 mice) and PCP-treated mice (n = 12 mice) for all variable delays (5, 15, and 30 sec). Note that the x-axis starts at 50%, the expected chance response accuracy. (I) No group differences in latency to reach the pellet cup during the variable delay test were observed. **p < 0.01, ^#^*p* < 0.05; NS, no significance. Line graphs show mean ± standard error of the mean (SEM). Bar graphs show mean ± SEM.

Working memory was measured using a discrete paired-trial variable-delay task in T-maze according to a previous report [23] with slight modifications. All behavioral analyses were recorded using a CCD camera and video tracking software (ANY-maze ver. 4.99; Stoelting Co., USA) under approximately 50-lux illumination. Before all experiments, mice were acclimatized to the experimental site for 30 min. This task was conducted using a T-maze constructed from rigid 3-mm thick polyvinyl chloride. The main alley (50 × 10 × 15 cm) was connected to two side goal arms (30 × 10 × 15 cm). Three sliding guillotine doors, 20 cm high and 9.9 cm wide, were manually operated to keep the test mouse in the starting area (start box) or to block their entry into the left and/or right goal arm. Mouse body weight was reduced to 80%–85% of baseline by food restriction and maintained at this level throughout the experiment with food pellets (20 mg dustless precision pellets, Bio-Serv). Drinking water was available *ad libitum* in the home cage. The discrete paired-trial variable-delay task in T-maze was performed over four sessions: (1) adaptation, (2) forced-alternation training, (3) discrete paired-trial delayed alternation training, (4) variable delay test (See Fig 1A). In the adaptation procedure, mice were habituated to the T-maze over 2 days. Mice were first placed in the T-maze with their cage-mate for 10 min and then placed in the maze alone for 5 min each once daily. For initial adaptation to task runs, a food pellet was placed in a pellet cup at the end of each goal arm and the mouse was released. This procedure was continued until also mice reliably entered the goal arms to eat pellets. This adaptation procedure was followed by the forced-alternation training, in which mice had to enter one goal arm and then the other in an alternating fashion to obtain pellets (Fig 1B). Briefly, the mouse was placed in the start box with either the left (L) or right (R) goal arm open and baited, and then allowed to obtain food pellets. If the mouse lingered for 2 min or more while traveling to or from the goal arm, it was gently pushed to initiate movement toward the goal arm as appropriate. The guillotine door of the start box was re-opened after a 5 sec intra-trial interval (delay), and mice were allowed to run into the maze arm which was in the opposite direction to that visited beforehand. Subsequently, they were removed from the maze and put into a holding cage, which was placed adjacent to the maze. This forced-alternation training trial was repeated at 10 times in a pseudorandom sequence manner (e.g. RLRRLRLLRL), at a 40 sec inter-trial interval. These sessions were run daily for 4 consecutive days. The discrete paired-trial delayed alternation training consisted of a pair of forced and choice runs (Fig 1D). Mice were allowed to enter the opened arm to eat a food pellet, and then were moved back to start box. After a 5 sec delay, mice were allowed access either maze arm in the choice run. This session was also repeated at 10 times in a pseudorandom sequence with each trial separated by 40 sec inter-trial interval, and continued until a criterion of 80% correct responses on three consecutive days was achieved. Mice that failed to reach this criterion after 14 days of this discrete paired-trial delayed alternation training were eliminated from the present study. Beginning the day after reaching the criteria, mice were treated with saline or PCP 10 mg/kg each day for 14 consecutive days. After a 4-day withdrawal period, they were subjected to the variable delay test (Fig 1G). Mice were given 4 trials at each delay (5, 15, and 30 sec) presented in a random order at 40 sec inter-trial intervals. A total of 12 trials for each delay were run over the 3 consecutive days. Percent correct responses were calculated as a performance index for working memory during training and test sessions.

### Brain-wide c-Fos mapping after working memory task

Brain sampling was performed at two time points according to the temporal pattern of the c-Fos protein induction that peaks within the range of 90–120 min after stimulus onset [26]. Either immediately or 90 min after completion of the T-maze task, the mice were anesthetized with sodium pentobarbital (100 mg/kg, i.p.) and xylazine (10 mg/kg, i.p.), and transcardially perfused with 0.1 M phosphate buffer (PB) containing 4% paraformaldehyde (PFA) plus 15% saturated picric acid. Brains were post-fixed with the same fixative overnight at 4°C, transferred to 15% sucrose in 0.1 M PB, and then immersed in 30% sucrose in 0.1 M PB for cryoprotection. Coronal sections (30-μm thick) were cut with a cryostat and stored at −30°C in a solution containing 30% (v/v) ethylene glycol, 30% (v/v) glycerol, and 0.1 M sodium PB, until use.

We conducted brain-wide c-Fos mapping in the following brain regions: prelimbic (PL) and infralimbic cortex (IL) of medial PFC (mPFC), anterior cingulate cortex (ACC) (1.98 to 1.70 mm from bregma), dorsomedial striatum (DMS), dorsolateral striatum (DLS) (anterior: 1.70 to 1.18 mm, medial: 0.98 to 0.62 mm, posterior: 0.50 to 0.14 mm from bregma), thalamic paraventricular (PVT), mediodorsal (MD) and reuniens (RE) regions, dentate gyrus (DG), CA1, and CA3 regions of dorsal hippocampus (dHPC) (−1.46 to −1.82 mm from bregma), ventral CA1 (vCA1)/subiculum of the ventral hippocampus (vHPC), dorsal substantia nigra pars compacta (dSNC) and medial SNC (mSNC), interfascicular nucleus (IF), and ventral tegmental area (VTA) medial (mVTA) and lateral (lVTA) regions (−3.28 to −3.52 mm from bregma).

Eight to twelve sections were used in each brain regions. Free-floating sections were rinsed with Tris-buffered saline (TBS) containing 0.1% Tween 20 (TBST) and incubated with 3% H_2_O_2_ in TBS to quench intrinsic peroxidase activity. The sections were rinsed with TBST, incubated with TBS containing 5% normal goat serum and 0.3% Triton X-100 for 1 h at room temperature (RT), and then incubated with the following primary antibodies overnight at 4°C: rabbit anti-c-Fos polyclonal antibody (1:2000, sc-52, Santa Cruz Biotechnology) and mouse anti-tyrosine hydroxylase antibody (1:2000, MAB318, Merck Millipore). Immunolabeled sections were rinsed with TBST and incubated with goat HRP-conjugated anti-rabbit IgG (1:500, ab6721, abcam) and goat Alexa 488-conjugated anti-mouse IgG (1:500, A11029, Molecular Probes) for 1 h at RT. After washing with TBST, sections were incubated for 5 min with Cy5-conjugated Tyramide (Tyramide signal amplification (TSA) Plus Cyanine 5 Kit, PerkinElmer, USA) made by diluting TSA stock solution 1:50 in 1 × Amplification Diluent. After washing, sections were mounted on glass slides using Vectashield Mounting Medium with 4', 6-diamidino-2-phenylindole (DAPI, H-1200, Vector Laboratories). To identify laminar cytoarchitecture in the cerebral cortex, sections adjacent to those used for c-Fos immunostaining were used for Ctip2 staining (1:500, ab18465, abcam) as a marker for cortical layer 5 (and 6) and Foxp2 staining (1:2000, ab16046, abcam) as a marker for cortical layer 6. Layer 2–3 was determined according to boundaries between the molecular layer 1 and Ctip2-immunoreactive layer 5.

### Image acquisition and data analysis

Acquisition of fluorescence images and image analyses were conducted by an experimenter blind to test conditions. For c-Fos mapping, fluorescence images were acquired using a fluorescence microscope (BZ-X700, Keyence) with 10 × objective lens to cover entire regions of the brain sections. All histological sections were scanned in multiple z-planes in 5-μm intervals through a full-focus image-processing module, and captured images were computationally unified using the full-focus image stitching function in BZ-X700. Each region of interest (ROI) was identified and clipped using 1) a mouse brain atlas [27], 2) fluorescence images of Ctip2 and Foxp2 immunostaining for layer-specific analysis in the frontal cortex and 3) the ROI manager function in ImageJ (ver. 1.49). Quantification of c-Fos+ cells (in mm^2^) was performed using “ROI manager,” “Subtract background,” “measure,” “threshold,” and “analyze particles” functions of ImageJ.

### Statistical analysis

Statistical analysis was conducted using Microsoft Excel and SPSS software (ver. 24, IBM Japan). Group means were compared using Student’s t test, two-way analysis of variance (ANOVA) or two-way repeated measures ANOVA with Greenhouse-Geisser correction for repeated measures if necessary. Bonferroni *post hoc* test was conducted when appropriate.

## Results

### Working memory task in T-maze

In the forced alternation training, there was no significant difference in latency to eat the pellet between mouse groups scheduled to undergo chronic saline or PCP treatment, and both groups readily learned to run quickly through the maze to consume the food pellet (Fig 1C). There was a significant main effect of training day on latency to eat the pellet (F(1, 21) = 14.471, *p* < 0.01) but no significant group × day interaction (F(1, 21) = 0.670, *p* = 0.422), and no significant main effect of group on latency to eat the pellet. In discrete paired-trial delayed alternation training, there was no significant group difference in days to reach the performance criterion (saline: 6.7 ± 1.0; PCP: 7.6 ± 0.8; *t*_(21)_ = 0.672, *p* = 0.508) (Fig 1E) or in correct response rates (saline: 85.2 ± 0.9%; PCP: 88.1 ± 1.7%; *t*_(21)_ = 1.480, *p* = 0.153) (Fig 1F), suggesting that these two groups did not differ in either acquisition or working memory task performance before drug treatment. In the variable delay test following treatment, there was no significant group × delay interaction (F(1.533, 32.190) = 0.486, *p* = 0.570), but there was a significant main effect of delay on percent correct responses (F(1.533, 32.190) = 5.714, *p* < 0.05) and main effect of group on percent correct responses (F(1, 21) = 10.653, *p* < 0.01) (Fig 1H) indicative of a delay-independent working memory impairment in the chronic PCP-treated group (delays: 5 s = 70.8 ± 2.6%; 15 s = 69.4 ± 3.0%; 30 s = 60.4 ± 3.9%) compared to control mice (delays: 5 s = 84.1 ± 3.8%; 15 s = 75.8 ± 4.0%; 30 s = 68.9 ± 4.2%). There was also a main effect of day (F(1.166, 24.496) = 4.194, *p* < 0.05) on latency to reach the pellet cup. However, there was no significant group × day interaction (F(1.166, 24.496) = 0.236, *p* = 0.668) and no main effect of group (F(1, 21) = 0.021, *p* = 0.887) on latency to reach the pellet cup, suggesting that both chronic PCP-treated and chronic saline-treated mice remembered the task rule (Fig 1I).

### Brain-wide c-Fos mapping after working memory task

**Fig 2:**
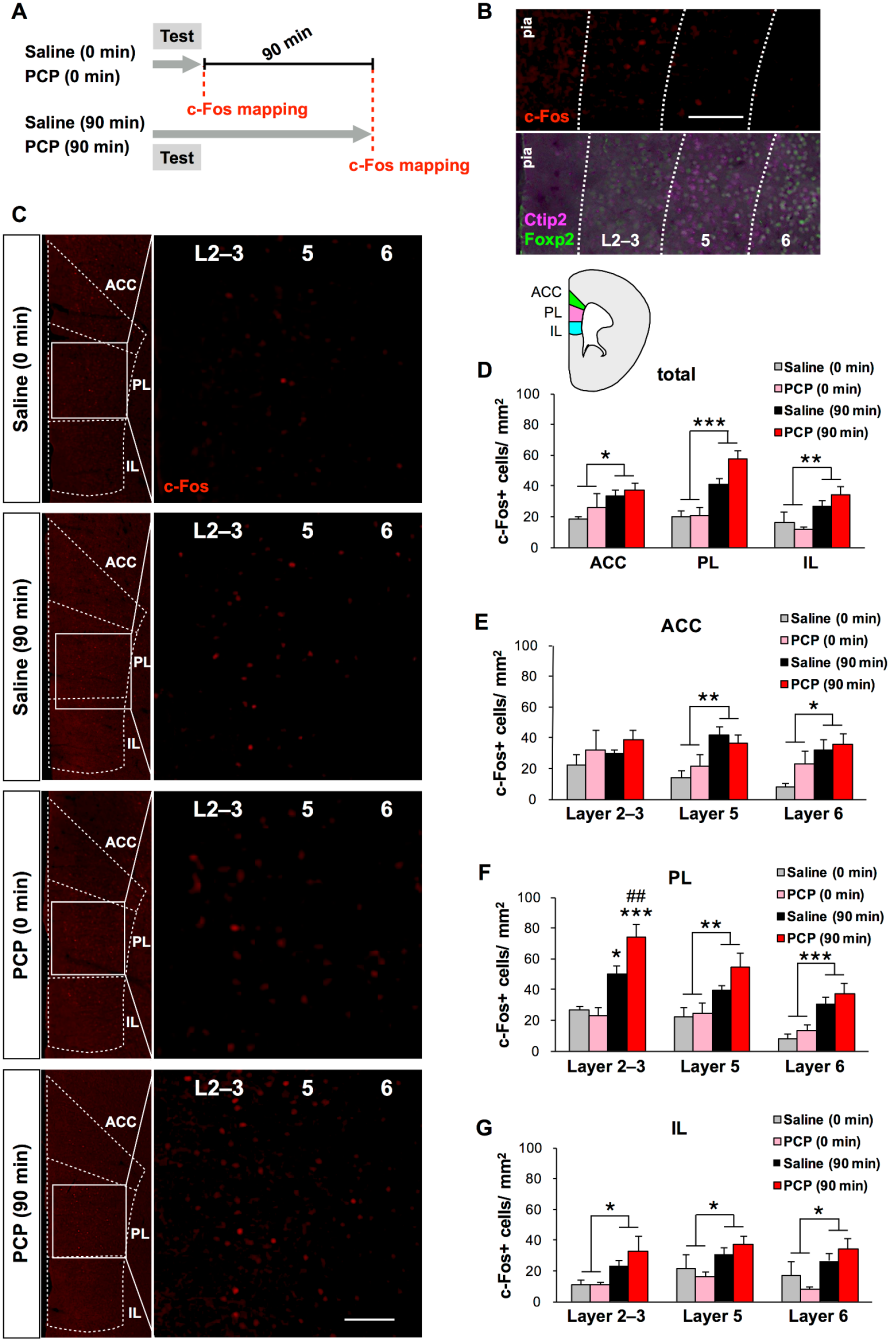
Quantification of c-Fos+ cells in the frontal cortex of chronic saline and PCP-treated mice. (A) Schematic illustration of brain sampling schedule for brain-wide c-Fos mapping after working memory task. The timing of sampling for c-Fos immunohistochemistry is indicated with the red dashed lines. (B) Representative c-Fos staining (red) in the PL (B, top). Representative double staining for a layer 5 (and 6) marker, Ctip2 (magenta), and a layer 6 marker, Foxp2 (green), in sections adjacent to those used for c-Fos immunostaining in the PL (B, bottom). Scale bar, 100 μm. (C) Fluorescence microscopy images of c-Fos staining (red) in the frontal cortex of chronic saline- or PCP-treated mice. Scale bars, 100 μm. (D) Quantification of the total number of c-Fos+ cells in the ACC, PL, and IL of chronic saline- (0 min: n = 4, 90 min: n = 5) and PCP-treated mice (0 min: n = 4, 90 min: n = 5). (E–G) Quantitative laminar-specific c-Fos mapping in the ACC (E), PL (F) and IL (G). *p < 0.05, **p < 0.01, ***p < 0.001 vs 0 min, ^##^*p* < 0.01 vs saline (90 min). Graphs show mean + SEM.

To determine brain regions relevant to working memory performance, we applied two-way ANOVA to disentangle the effect of the types of chronic treatment (PCP vs saline) and sampling time (90 min vs 0 min) on the number of c-Fos+ cells in the brain (Fig 2A). We determined cortical layer boundaries by performing immunostaining of the layer markers, Ctip2 and Foxp2 (Fig 2C). Across layers 2–6 of the ACC (F(1, 14) = 5.919, *p* < 0.05), PL (F(1, 14) = 27.000, *p* < 0.001), and IL (F(1, 14) = 9.386, *p* < 0.01) regions, there was a significant main effect of the working memory effort relative to basal conditions on the total numbers of c-Fos+ cells (Fig 2D), although there were no significant effects of treatment groups or their interaction with sampling time. When examined layer by layer, there were no significant effects of interaction between the drug treatment and sampling times on the number of c-Fos+ cells in layers of ACC, PL and IL (Fig 2E–2G), except for the layer 2–3 in the PL where there was a significant interaction. *Post hoc* analysis revealed that the number of working memory effort-elicited c-Fos+ cells was significantly higher in the chronic PCP group than in the chronic saline group in layer 2–3 of the PL (*p* < 0.01), and that the number of c-Fos+ cells was significantly higher at 90 min after the working memory effort relative to basal condition (at 0 min after the working memory effort) in the chronic PCP group (*p* < 0.001) (Fig 2F). In contrast, there were significant main effects of sampling time on the number of c-Fos+ cells in the layers 5 and 6 of the PL (layer 5: F(1, 14) = 12.517, *p* < 0.01, layer 6: F(1, 14) = 23.303, *p* < 0.001), ACC (layer 5: F(1, 14) = 13.657, *p* < 0.01, layer 6: F(1, 14) = 7.300, *p* < 0.05), and IL (layer 2–3: F(1, 14) = 7.632, *p* < 0.05, layer 5: F(1, 14) = 6.624, *p* < 0.05, layer 6: F(1, 14) = 8.340, *p* < 0.05). These results suggest a layer-specific difference in PL activity between chronic PCP- and saline-treatment groups during working memory task.

**Fig 3:**
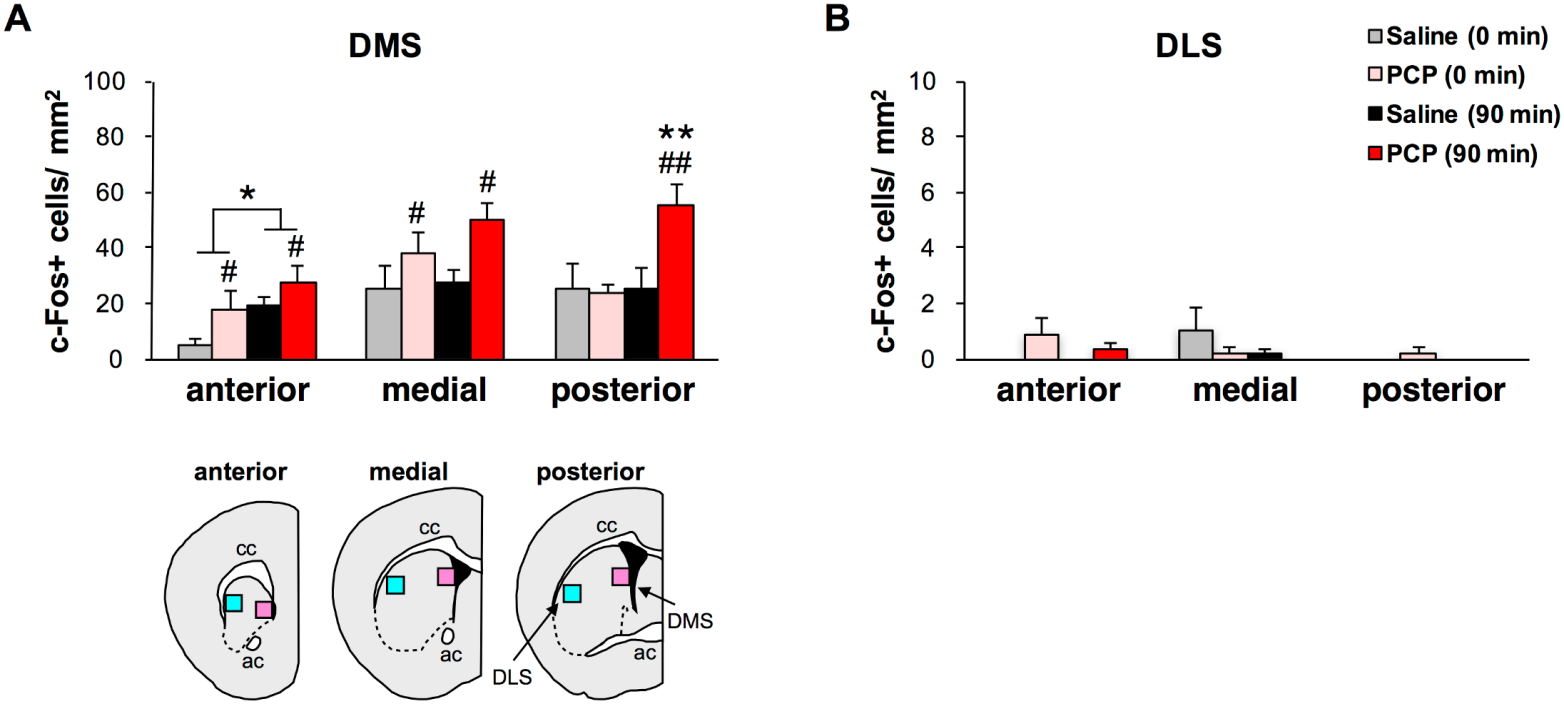
Quantification of c-Fos+ cells in the striatum of chronic saline- and PCP-treated mice. Quantification of c-Fos+ cells in the DMS (A) and DLS (B) of chronic saline- (0 min: n = 4, 90 min: n = 5) and PCP-treated mice (0 min: n = 4, 90 min: n = 5). *p < 0.05, **p < 0.01 vs 0 min, ^#^*p* < 0.05, ^##^*p* < 0.01 vs saline. Graphs show mean + SEM.

In the striatum, there were no significant drug treatment × sampling time interactions on the number of c-Fos+ cells in the DMS and DLS, except for the posterior part of the DMS (Fig 3A and 3B). In the posterior part of the DMS, *post hoc* analysis revealed that the number of working memory effort-elicited c-Fos+ cells was significantly higher in the chronic PCP group than in the chronic saline group (*p* < 0.01), and the number of c-Fos+ cells was significantly higher at 90 min after the working memory effort relative to basal condition in the chronic PCP group (*p* < 0.01) (Fig 3A). In the rest of the DMS, other than posterior part, there were significant main effects of sampling time (anterior: *p* < 0.05), and drug treatment (anterior: *p* < 0.05, medial: *p* < 0.05). In contrast, there was no significant main effect of drug treatment and sampling time in all segments (anterior to posterior) of the DLS (Fig 3B).

**Fig 4:**
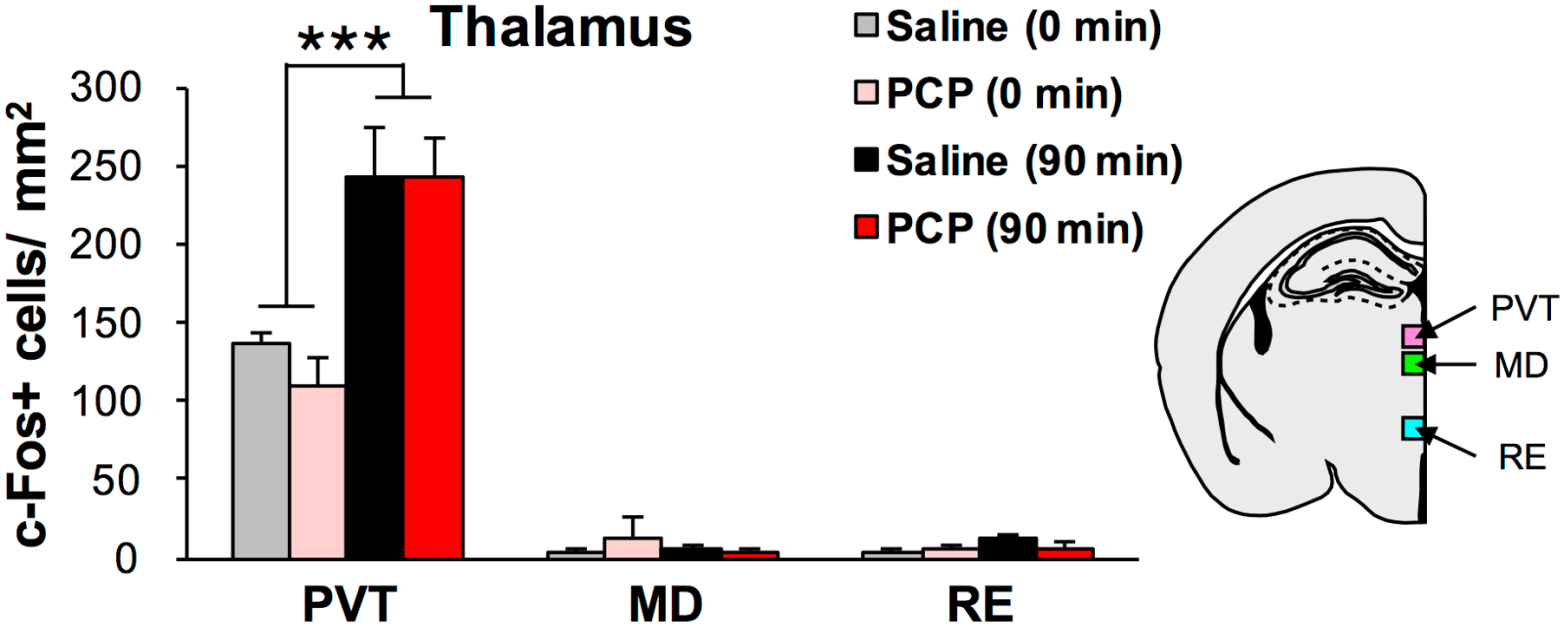
Quantification of c-Fos+ cells in the thalamus of chronic saline and PCP-treated mice. Quantification of c-Fos+ cells in the PVT, MD and RE of the thalamus of chronic saline- (0 min: n = 4, 90 min: n = 5) and PCP-treated mice (0 min: n = 4, 90 min: n = 5). ***p < 0.001 vs 0 min. Graphs show mean + SEM.

We observed that there were no significant drug treatment × sampling time interactions on the number of c-Fos+ cells in all regions (PVT, MD and RE) of the thalamus, and significant main effect of sampling time on the number of c-Fos+ cells in the PVT (*p* < 0.001) (Fig 4).

**Fig 5:**
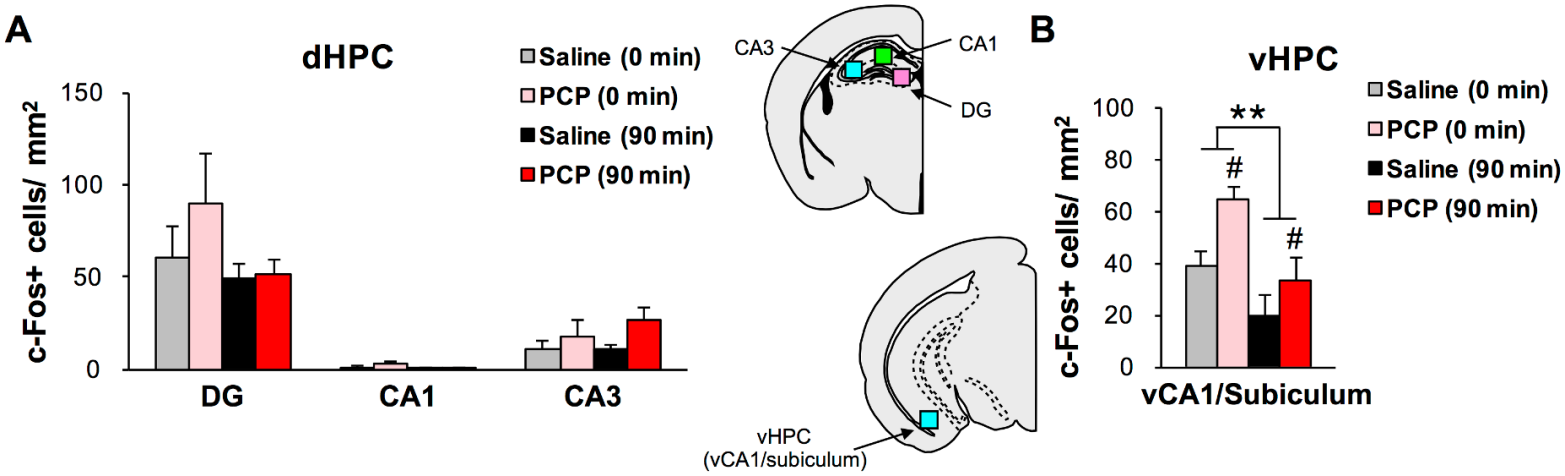
Quantification of c-Fos+ cells in the hippocampus of chronic saline- and PCP-treated mice. (A and B) Quantification of c-Fos+ cells in the DG, CA1, and CA3 of the dHPC (A) and vCA1/subiculum of the vHPC (B) of chronic saline- (0 min: n = 4, 90 min: n = 5) and PCP-treated mice (0 min: n = 4, 90 min: n = 5). **p < 0.01 vs 0 min, ^#^*p* < 0.05 vs saline. Graphs show mean + SEM.

In the hippocampal regions, c-Fos expression levels were comparable regardless of drug treatment (presence or absence of PCP treatment), or working memory effort (sampling time) in the dorsal part of the hippocampus (Fig 5A). In contrast, we observed differential changes in c-Fos expression in the vCA1/subiculum of the ventral hippocampus, where there were significant main effects of both drug treatment (*p* < 0.05) and sampling time (*p* < 0.01) despite no significant drug treatment x sampling time interaction (Fig 5B). In the vCA1/subiculum, chronic PCP treatment showed elevated c-Fos expression regardless of working memory effort, whereas the number of c-Fos+ cells was significantly lower at 90 min after the working memory effort relative to basal condition both in the chronic saline and PCP groups.

**Fig 6:**
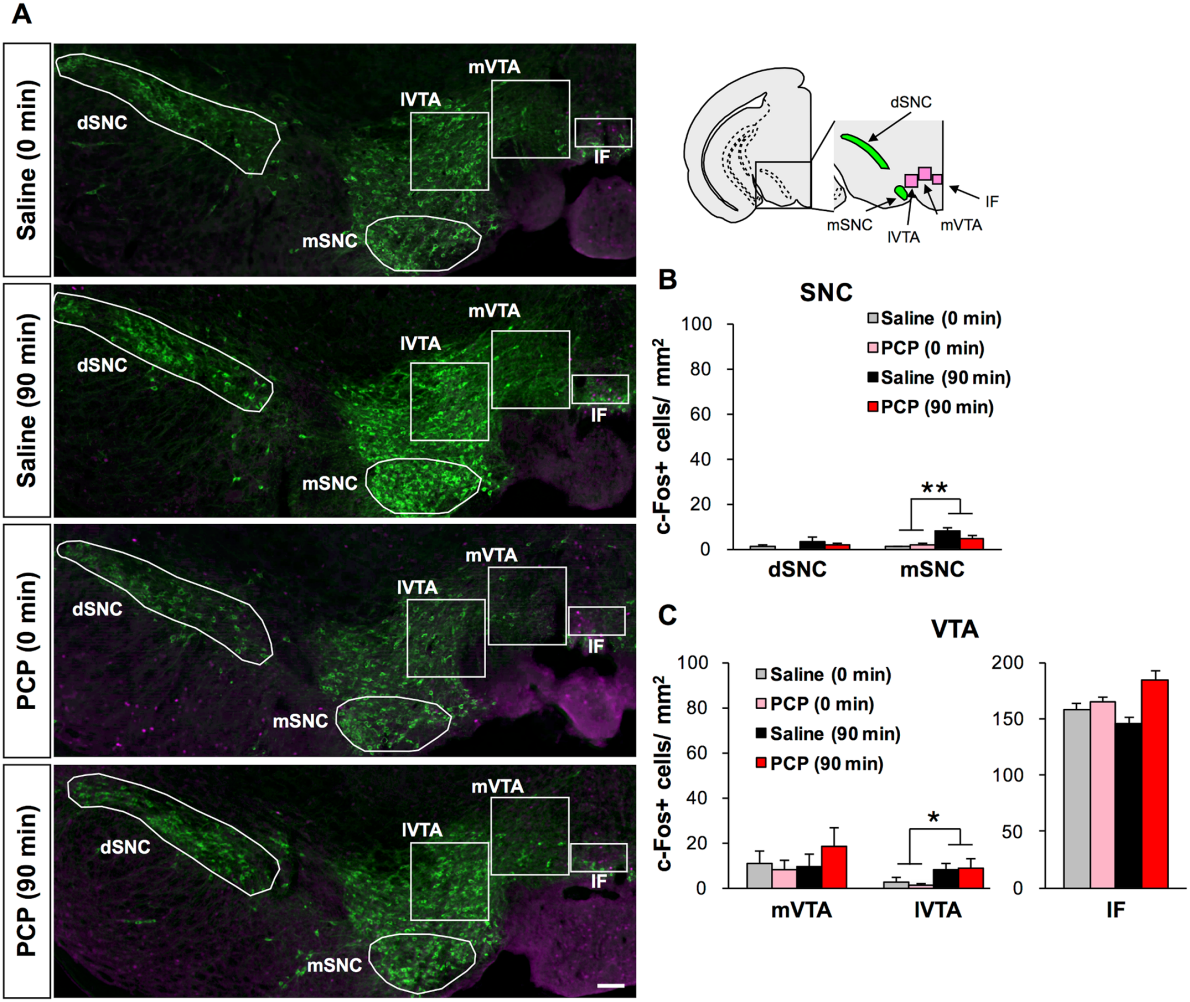
Quantification of c-Fos+ cells and overlap of c-Fos and tyrosine hydroxylase immunoreactivities in the ventral tegmental area and substantia nigra of chronic saline- and PCP-treated mice. (A) Representative full-focus images showing c-Fos and TH immunoreactivities in the VTA and SNC of chronic saline- and PCP-treated mice. Scale bar, 100 μm. (B and C) Quantification of c-Fos+ cells in the dSNC and mSNC of the SNC (B) and mVTA, lVTA and IF of the VTA of chronic saline- (0 min: n = 4, 90 min: n = 5) and PCP-treated mice (0 min: n = 4, 90 min: n = 5) (C). *p < 0.05, **p < 0.01 vs 0 min. Graphs show mean + SEM.

No changes in c-Fos expression were observed in the dSNC, mVTA or IF, whereas working memory effort significantly increased c-Fos expression in the mSNC (*p* < 0.01) (Fig 6B) and lVTA (*p* < 0.05) (Fig 6C) of both the chronic saline and PCP group. To address whether the working memory task differentially affects the activity patterns of dopamine neurons, we examined SNC and VTA c-Fos+ neurons using co-immunolabeling with TH, a marker of dopaminergic neurons in these regions (Fig 6A). c-Fos expression was comparable regardless of drug treatment, or working memory effort in the dSNC, mSNC, mVTA and IF, and slightly increased by working memory effort in the lVTA (*p* < 0.05) (S1 Table).

## Discussion

In the present study, we used the discrete paired-trial variable-delay task in T-maze [23] to assess working memory deficits in the chronic PCP rodent model of schizophrenia. The Cognitive Neuroscience Treatment Research to Improve Cognition in Schizophrenia (CNTRICS) meeting has recommended several conditions for behavioral paradigms to recapture the aspects of working memory impairment in human neuropsychiatric conditions [28]. In this regard, the discrete paired-trial delayed task and the variable-delay task in T-maze, which is equivalent to the delayed non-match to position task used for human study, have been ranked highly for assessment of goal maintenance, an essential factor of working memory according to the CNTRICS.

The primary aims of this study were to (1) evaluate the working memory performance in mice following chronic PCP administration and to (2) identify the neuronal circuitry relevant to task performance using c-Fos mapping. To our knowledge, this is the first report to investigate these study aims. The main findings are summarized as follows. (1) Chronic treatment with the dose regimen of PCP in the present study induced delay-independent impairments in working memory in mice. (2) The working memory effort resulted in greater numbers of c-Fos+ cells in layer 2–3 of the PL in the chronic PCP groups relative to chronic saline groups, whereas there was a main effect of working memory effort relative to basal conditions in inducing significantly larger numbers of c-Fos+ cells in other layers of the PL, layers 5 and 6 of the ACC and layers 2–3, 5, and 6 of the IL of the mPFC, anterior DMS and PVT regardless of the type of chronic drug treatment (PCP vs saline groups).

Our behavioral data show that the main effect of chronic PCP treatment in lowering correct response rates across three delays (5, 15, and 30 s) is independent of the delay. This is consistent with the report of Chudasama and colleague that lesion of the PL induced delay-independent impairment in a delayed non-matching to position task using operant chamber in rats [29]. These lines of evidence are consistent with a meta-analysis of working memory impairments in patients with schizophrenia that reported that working memory impairments are not delay-dependent and do not worsen with delays longer than at least 1 s [30]. However, there are some inconsistencies regarding the extent to which rodents performance worsens on this task after chronic PCP treatment, with some studies reporting that chronic PCP treatment impairs variable-delayed alternation task [31–33], while others have found no such effect [34]. This discrepancy among studies may be attributable to the different PCP dose regimens, withdrawal periods, intra-trial intervals (delays), inter-trial intervals (ITIs) and strains of mice and rats used. We argue that chronic PCP treatment using our dose regimen is a valuable strategy for exploring potential target regions relevant to working memory impairments and treatments in animal models of schizophrenia.

Multiple behavioral paradigms, including operant-, maze- and touchscreen-based tasks, combined with various lesions, pharmacological and genetic manipulations, and electrophysiological recording have shown that the rodent mPFC plays an important role in working memory [28]. Indeed, electrophysiological studies have shown changes in neural activity in mPFC during the delay period in such tasks [35, 36]. The mPFC can be divided into four subdivisions in rodents: the infralimbic (IL), prelimbic (PL), dorsal and ventral anterior cingulate, and medial precentral (also known as second frontal area (Fr2)) cortices [37, 38]. Although most previous studies on working memory in rodents did not distinguish between PL and IL, spatially selective manipulation studies have revealed that PL appears to be critical for working memory [29, 39].

Our two-way ANOVA aimed at disentangling the effect of working memory effort and chronic PCP exposure on the number of c-Fos+ cells revealed that working memory efforts exerted significant main effects on the increased number of c-Fos+ cells in several regions of the brain in mice regardless of chronic treatment types. Greater c-Fos expression was also reported in the mPFC of mice subjected to a working memory version of the eight-arm radial arm maze test [40]. Collectively, the robust effect of working memory effort on the number of c-Fos+ cells in a wide range of brain areas demonstrates that a comparison based on time elapsed after working memory effort (90 min vs 0 min) is a valid strategy. Of note, the layer 2–3 of the PL is remarkable, in that only in this layer, a significantly larger number of working memory-elicited c-Fos+ cells was found in the chronic PCP-treated mice than in the chronic saline-treated mice. This finding suggests that the layer 2–3 of the PL is central to the response to working memory effort that is impaired in a delay-independent manner in the chronic PCP group.

The PL, IL and ACC have distinct input and output projections and subserve distinct functions. The PL receives main afferent inputs from the contralateral PL as well as orbital and agranular insular cortex, CA1 and subiculum of the vHPC, claustrum, basal nuclei of the amygdala, thalamic PVT, MD, and RE, and monoaminergic nuclei including the VTA [41]. It seems likely that the mPFC (which includes the PL) does not act alone during working memory tasks, as is evident in recent studies showing that other brain regions, including the DMS, MD, and RE of the thalamus, and hippocampus have been implicated in working memory in concert with their interactions with mPFC. The PL sends efferents to the DMS, ventral striatum, claustrum, basolateral amygdala, lateral hypothalamus, RE and MD thalamus, VTA, dorsal raphe and, periaqueductal gray [38, 42, 43]. We also found a significantly higher number of working memory-elicited c-Fos+ cells in the posterior parts of the DMS in chronic PCP-treated mice compared with control mice. Several lines of evidence, including lesion studies [44], inactivation studies, and electrophysiological measurements [45], indicate that the DMS is involved in the performance of behavioral paradigms that require working memory. DMS receives direct inputs from the PL and ACC [38, 43]. Moreover, the posterior DMS, in which working memory-elicited c-Fos expression was significantly larger in chronic PCP-treated mice than in the saline-treated group, is primarily innervated by neurons in the layer 5 and 6 of the PL [46]. This direct anatomical connection suggests that the DMS and PL may cooperatively contribute to working memory, warranting further study of the neural circuitry connecting these regions in a model of working memory.

The other brain area that warrants greater attention regarding schizophrenia-related working memory deficits is the hippocampus, as suggested by the classical inactivation experiments using delayed non-matching to position tasks [47, 48]. Anatomical studies demonstrated that the PL and IL receive direct monosynaptic connections from the vHPC including the subiculum [41]. Evidence suggests that transfer of spatial information processed in the hippocampus to the mPFC is crucial for spatial working memory task performance, as is the case for the delayed non-match to position and delayed alternation paradigms [49–52]. A more recent study showed that optogenic silencing of vHPC to mPFC connectivity disrupts the encoding of goal location information in the mPFC during the sample phase of a delayed non-match to place task [53], whereas another study indicated a role for the vHPC in the choice phase [54]. Furthermore, Tamura et al. reported that mPFC neurons phase-locked most strongly to gamma rhythm in the vHPC during encoding that possessed more spatial representation about the goal location during choice phase [55]. However, a few studies on rodent spatial working memory have examined the effect of working memory effort on hippocampal function in terms of neural mapping. Our finding that the working memory effort was a main factor in decreasing the number of c-Fos+ cells in the CA1/subiculum of the ventral hippocampus may reflect a disrupted connectivity between the hippocampus and mPFC during task performance in the chronic PCP groups. The chronic PCP-induced increase in the number of c-Fos+ cells in the CA1/subiculum of the ventral hippocampus is another area of interest. For example, basal *c-fos* mRNA expression was higher in the posterior hippocampus of subchronic PCP-treated mice after 4 days withdrawal [56]. CA1/subiculum of the ventral hippocampus has been reported to be highly vulnerable to high demand of metabolism in high-risk patients who are prone to develop psychosis, and in rodents treated with chronic ketamine [57]. Therefore, elevated c-Fos protein expression of vCA1/subiculum observed in the present study may reflect hyperactivation in basal state in this region of chronic PCP-treated mice which is consistent with the two above-mentioned reports.

RE and PVT represent ventral and dorsal structures in the midline nuclei of the thalamus, respectively [58]. mPFC is also indirectly connected to the dHPC via the thalamic RE [52]. Of note, a role for the RE in a delayed non-match to position task was reported [59]. Although the present data revealed no change in the number of c-Fos+ cells in the RE associated with working memory effort, dHPC inactivation, but not mPFC inactivation, was reported to impair working memory and also increase choice response latencies [49]. Further study is required to elucidate the distinct role of the HPC across the dorsal to ventral axis in working memory processing that is finally executed by the mPFC. PVT is known to have a strong reciprocal connection with mPFC, and to send an output to the amygdala and nucleus accumbens [60]. Although involved in affective and reward behaviors, the role of the PVT in working memory remains unknown, warranting further study [58].

The VTA has reciprocal anatomical connections with cortical and subcortical areas, and is thought to play a key role in working memory as evaluated by delayed alternation [61] and delayed non-match to sample tasks [62]. The present study found that working memory effort increased the number of c-Fos+ cells in the mSNC and lVTA for both the chronic PCP- and saline-treated groups, but caused no group differences in c-Fos+ cell numbers in any of subregions of the VTA and SNC. On the other hand, our finding that there was no main effect of prior chronic regimens (PCP vs saline) on the number of c-Fos+ cells across subregions of the midbrain dopaminergic neurons suggests that chronic PCP exposure is unlikely to alter the dopaminergic reward system.

One limitation of the present study is that, given that various features of working memory, such as goal maintenance and interference control, shape the discrete paired-trial variable-delay task [28], it is difficult to disentangle the involvement of these features by c-Fos immunocytochemistry alone because of its low temporal resolution. Another confounder that may have affected the interpretation of results is that in most regions, we counted the number of c-Fos+ cells without colabeling for neurotransmitters or their synthetic enzymes. Further qualitative and quantitative analyses of cell types or neural pathways activated by working memory effort are warranted.

## Conclusion

In the present study, we demonstrate that mice chronically treated with PCP show impaired spatial working memory as evaluated by the discrete paired-trial variable-delay task in T-maze. Furthermore, working memory effort results in much higher numbers of c-Fos+ cells in the prelimbic cortex (layer 2–3) in the chronic PCP-treated mice than in chronic saline-treated mice. The strength of the present study is the identification of brain regions relevant to working memory impairment in the PCP model. Despite the lower temporal resolution of c-Fos mapping and our currently limited understanding of the anatomical associations among the neural circuits within these regions, further pathway-specific and temporally precise analyses are warranted. Such studies may lead to the identification of novel therapeutic targets for schizophrenia patients.

## Supporting information

S1 Table Quantification of c-Fos and TH double-positive cells in the SNC and VTA. (DOCX)
