# Supplementary material for: Abnormal neural activation patterns underlying working memory impairment in chronic phencyclidine-treated mice
Source: PLoS One. 2017 Dec 18;12(12):e0189287. doi: 10.1371/journal.pone.0189287 (PMC5734723; doi:10.1371/journal.pone.0189287)
Supplement: S1 Table — (DOCX) [file pone.0189287.s001.docx]

| **S1 Table. Quantification of c-Fos and TH double-positive cells in the SNC and VTA** | | | | | |
| --- | --- | --- | --- | --- | --- |
| **Brain area** |  | **Saline** | | **PCP** | |
| Sampling time |  | 0 min | 90 min | 0 min | 90 min |
| Cell Counts (/ mm^2^) |  |  |  |  |  |
| Substantia nigra pars compacta | dSNC | 0.00 ± 0.00 | 0.08 ± 0.08 | 0.00 ± 0.00 | 0.05 ± 0.05 |
|  | mSNC | 0.00 ± 0.00 | 0.15 ± 0.03 | 0.03 ± 0.03 | 0.20 ± 0.15 |
| Ventral tegmental area | mVTA | 0.16 ± 0.08 | 0.23 ± 0.17 | 0.09 ± 0.06 | 0.28 ± 0.13 |
|  | lVTA | 0.00 ± 0.00 | 0.18 ± 0.06 | 0.00 ± 0.00 | 0.33 ± 0.16 |
|  | IF | 0.69 ± 0.34 | 0.75 ± 0.24 | 0.38 ± 0.22 | 0.5 ± 0.18 |

Values represent average ± SEM.
